# Counter Practice

**Published:** 1862-07

**Authors:** 


### Art. IV.—COUNTER PRACTICE.

Among the inconveniences incidental to the fulness of English liberty, there is perhaps no more prominent professional grievance than that which we have selected to give a title to this article. The Apothecaries' Act of 1815, by its tendency to degrade the medical practitioner into a druggist, had also the contrasted effect of elevating the druggist towards the status of a medical practitioner. We are acquainted with a populous town in one of the midland counties, where dwelt, and practised for many years, a surgeon of great and deserved repute. His period of activity was prior to the invention of broughams; and, for a long space, he was driven in a gig by one old and faithful servant. A poor neighbour, afflicted with some serious illness, and unable to pay the master's charges, bethought him of applying for professional assistance to the man. It was impossible, he argued, that Thomas should have driven Mr. Healingart for so long a time, without learning a great deal. Thomas, nothing loth, accepted the confidence reposed in him, was content with smaller fees than those demanded by his master, and, during the progress of the case, actually showed his full approval of the principle involved, by seeking a consultation with the groom of a neighbouring physician. The patient died; but Thomas, nothing daunted by an accident which was of frequent occurrence among his master's patients also, made his "first case" serve as the foundation for what eventually became a large and lucrative practice.

Now, the public have trusted druggists much on the same principle that influenced the too confiding patient to trust in the surgeon's charioteer. Surely, a man who has opportunities of smelling rhubarb every day must inhale, together with its balmy odours, a capacity for curing fever, or for detecting inflammation of the lungs. Surely, the frequent contact of senna with the fingers must convey a knowledge of the nature and causes of infantile convulsions; and the habitual perusal of prescriptions must afford information about that mysterious *x*, the "constitution" of the patient for whom the draught or mixture is intended. Such, at least, is the line of argument usually adopted by those who are, or fancy themselves, only a "little poorly." The medical profession, as a body, by setting up "physic" before the populace as an idol to be worshipped, has been compelled to divide the spoils of the devotees with the humbler servants at the shrine, and to witness the successes of a large body of dealers in doctor's stuff, who, like Thomas, have at least been near the rose, and who, also like him, appeal to that deeply-rooted sentiment of our nature which was described by the late Sir Robert Peel as an "ignorant impatience of taxation." Thomas and the druggists are cheap, while doctors, relatively to the means of the poor, are often very dear.

For many years past, under the influence of these causes, nearly all druggists have been accustomed to drive a lucrative trade in what is euphoniously called "Counter Practice;" and only those who have paid some attention to the matter can form any just idea of the extent to which this system has been carried on, or of the numerous classes of society to which it has spread. Among the poor, the druggist is the universal first resort in the case of any illness that does not appear immediately to threaten life; and even among the wealthier classes, his shop is greatly frequented, and his advice highly valued. The source of remuneration is the sale of medicine, at prices varying from a few pence for the poor, to more ordinary rates of charge. A little child, with cough and fever, will be treated to a "calomel powder," and a mixture with some saltpetre and a little squills, put into the mother's hair-oil bottle. For this, twopence or threepence will be charged; and the dose repeated daily until twopences fail, or until the natural close of neglected pneumonia is manifestly at hand; in either of which cases the parish doctor will be strenuously recommended. For the dyspeptic ailments of wealthier customers, there are medicines prepared from the prescriptions given by some popular physician to some county magnate; it being certain that the stomach of an alderman cannot continuously rebel against the dose that has soothed the discomforts of a Lord-Lieutenant. Druggists, whose local connexions do not enable them to dispense such signal favours as these, to boast of the Duke's dinner-pills, or Sir Astley Cooper's mixture, rest upon mysterious knowledge of their own, and speak of "something" that they will "put up"—a something usually prompted entirely by the patient's own recital of symptoms, and arrived at by an association of ideas that it would be both curious and difficult to unravel.

Until lately, counter practice has been spoken of as an admitted evil, railed at by doctors as a robbery, defended by druggists only on the score of being profitable. Since the passing of the Medical Act, however, the Filmer of this "right divine to practise wrong" has appeared in the shape of the editor of the *Chemist and Druggist*, who comes forward as the champion of his brethren, and has obtained from Mr. Chitty, the barrister, an opinion in support of their privileges. The case and the opinion are as follows :—

"QUESTIONS.

"1. Has a Chemist, &c., a right to mix up at his shop, for a patient, a medicine which the Chemist considers fit and proper for the case?

"2. May he, if sent for, attend a patient for that purpose?

"3. In either case, is he entitled to recover the value of his medicines, if he supply the same on credit?

"4. Is there any criminal liability attaching to a Chemist, &c., who, in dispensing medicines to a casual customer, commits an error through ignorance of the patient's constitution; if the disorder which the regular medical attendant of the patient might have alleviated, prove fatal through the want of knowledge on the part of the Chemist of previous symptoms of the particular case?"

The following opinion has been received on these points :—

"OPINION.

"1. I am of opinion that a Chemist may, in his shop, so far prescribe for a customer as to advise with him as to the nature and quality and mode of application of the medicines which he is about to sell, and also as to which of his commodities will best suit the requirements of his customer. He may listen to his customer's statements as to the reasons for his wishing to become a purchaser, and may suggest to and advise him as to which of his commodities will be most suitable and beneficial to the customer, or may dissuade him from purchasing or taking that which a customer in his ignorance may have applied for. This advice is merely incidental to the sale and dispensation of the Chemist's wares and drugs, and cannot, of course, be made the subject of a charge.

"2. I should say that in no case can a Chemist attend upon a patient or customer at the house of the latter, for the purpose of giving him advice or of seeing him, so as to be able to form an opinion as to the mode in which the Chemist should prescribe for or supply his patient with medicines. Of course, a Chemist may go to his customer's house to take an order for specific goods from the customer, if the latter send for him, he being too unwell to go out, just as any other tradesman may do.

"3. In either case a Chemist may, of course, recover for goods sold by him on credit; he is no more compelled to deal for ready money only than is any other tradesman. But, in all cases, the Chemist must be simply a 'vendor of his goods,' in no way seeking to make a profit by the advice or recommendation given by him. It seems to me, upon the whole, that the system which is called 'counter practice' is perfectly legal.

"4. I am of opinion, that a Chemist who does no more than what I have suggested he may legally do, is no more liable to criminal proceedings than is any other practitioner. He makes no professions, does not hold himself out in a false position, and if he acts *bonâ fide* with ordinary skill and ability, not being guilty of gross negligence, he stands in about the same position as any other medical practitioner. I have not thought it necessary to cite any authorities; but I believe the history of all the professions, and all the cases on the subject, are collected and referred to in 'The Attorney-General *v.* The Royal College of Physicians,' 30 Law, J., Chanc. 757. TOMPSON CHITTY.

"2, Essex-court, Temple, November 12, 1861."

We cannot but think that Mr. Chitty's opinion, by defining precisely the nature and magnitude of the evil involved in "counter practice" will pave the way for efforts at its extinction. Before expressing our feeling about the amount of mischief done by the system, we are bound to state our conviction that the Apothecaries' Act and its licentiates are mainly answerable for it; the druggists themselves being so only in a very subordinate degree. The apothecaries, in entering upon new duties should have abandoned their old ones; and in assuming the status of practitioners, should have ceased to act as dispensers of medicine.

The effect of producing a class of men who strove to combine both functions was, necessarily, to invade the peculiar province of the druggist to a very great extent; and to take away, as far as the supply of physic was concerned, the bulk of his custom. The counter practice, with all its evils, is but a feeble attempt at retaliation for this great injury; and not, as many general practitioners imagine, an entirely new and original aggression upon themselves.

The first step, therefore, towards the attainment of a better state of things must be, we apprehend, the absolute abandonment of dispensing by medical practitioners; and the second, the use of such means as may bring an authorized prescription within the reach of the poor. If these objects could be attained, the inducements to "counter practice" would be removed, inasmuch as druggists would certainly decline to assume medical responsibilities, when such assumption had entirely ceased to be a matter of profit.

Reserving for the present a more detailed consideration of the above desiderata, we will glance at the effects of counter practice upon the public, and also upon the profession.

With regard to the first, we will select three classes of disease —namely, purulent ophthalmia, diarrhœa, and inflammations of the lungs, as occurring among the infants and young children of the poor.

To purulent ophthalmia in early infancy, or to asthenic keratitis during childhood, may be ascribed a very large proportion of the cases of blindness to be met with among the lower orders of the populace. No disorders are more certainly amenable to timely treatment; and in none does the period for that treatment more frequently pass away, while the children are being physicked across the counter by an ignorant druggist. The statistics of an eye infirmary in a provincial town show seventy-two cases of infantile purulent ophthalmia during a single year. Of these, sixty-one children were brought to the institution at the commencement of the disease; and all were cured. The remaining eleven were brought with sloughing corneæ. They had all been under the treatment of druggists; and the eyes were either destroyed or irreparably damaged in every case.

In the same place, the mortality from diarrhœa was so great for several successive years, as to call for special inquiry from the Health Department of the Privy Council. The evidence of the parish medical officers went to show that, in cases of infantile diarrhœa, usually directly traceable to improper feeding, druggists were customarily first applied to, even by those parents who were able to obtain an order without difficulty; and that it was common to find the little creatures moribund, and semi-comatose from the opiate contained in the druggists' astringent mixtures.

With regard to inflammatory affections of the lungs, it is difficult to obtain a precise idea of the proportion of mortality that may be due to improper treatment. But when we consider that probably nine-tenths of all the cases occurring in towns, among the children of parents working for wages, are taken to a druggist at the beginning of the illness, and as his *out*-patients, often in inclement weather, undergo twenty-four or forty-eight hours of the saltpetre and squills, it becomes almost a marvel that any of them survive. In a town with a population of 60,000 souls, we have known the coroner hold three inquests upon such cases in the course of a single week; and in one of these, where the child had been carried backwards and forwards to the druggist for eight or ten successive days, always to receive a fresh supply of "the mixture as before," and at last to die whilst upon its journey home, the intelligent jury refused to concur in a censure upon the vendor of physic,—observing that "*if Mr. Pestle had known the child was dangerously ill,* of course he would have told the mother to take it to a doctor."

Besides these general illustrations, a recent trial has furnished us with a very striking and particular one. We extract from the *Times,* of May the 1st, the case of "Dungay *v.* Quiller," which may safely be left without comment to enforce the moral that it bears.

"DUNGAY *v.* QUILLER.

"Mr. M'Mahon and Mr. Gordon Allen were counsel for the plaintiff; Mr. Serjeant Parry and Mr. Lumly Smith for the defendant.

"This was an action for negligence.

"The plaintiff was a surveyor, and the defendant was a chemist and druggist in Sloane-square. In June last the plaintiff had a touch of the gout, and he went to the defendant's shop and saw his assistant, and told him he was recommended by the beadle to come there for some Smith's gout pills. The assistant put some pills in a box, and gave it to the plaintiff, and wrote a direction that one was to be taken twice a day. Plaintiff asked him if there was any colchicum in the pills. The assistant said there was not. He then asked if there was anything likely to give him cold, as he walked about in the wet. The assistant said there was not. Plaintiff asked him if they were good pills, and he said he believed they were. Plaintiff asked him if two did not do him good, whether he might take three a day. The assistant said he might. The plaintiff paid 8d. for the box, and went away. He then took two pills on that day, the 2nd of June. The next day he took two or three, and on the third and fourth days he took three. After he had taken the last of the pills he found himself very ill—great trembling in the limbs. He went to his doctor, Mr. Goodrich, who told him to go home directly. He was ill in London till September. He was for three weeks unconscious. On the 15th of June plaintiff's wife wrote to the defendant. He was afterwards sent to Margate, where he remained a fortnight. Had suffered from gout for many years. Had taken Bagster's pills, which had been recommended by his maid-servant, as they had done her father good. Smith kept the Star and Garter. The beadle told plaintiff he was obliged to leave off taking the pills, but they had done him good in the first place, but he recommended plaintiff to try them. The beadle was now down below. During the illness of the plaintiff the pill-box was shown to the defendant, and he was told he had made a mistake. He was asked how much calomel there was in each pill. The assistant said about two-and-a-half grains. The defendant said, 'Oh, no; only one grain.'

"Mr. Francis Goodrich said he saw the plaintiff in the early part of June. He was in bed. His face much swollen, his tongue out of his mouth. He could not articulate. He was in a high state of salivation. He must have taken some preparation of mercury. He was ill for many weeks. It was not proper to give him mercurial doses at the rate of two grains a day. The plaintiff's health had suffered much and was permanently injured. His life was in danger. My bill would be about one guinea a week for ten weeks.

"Cross-examined.—I have known plaintiff for twenty years. He has suffered from chronic gout. I might have given him a blue pill.

"The defendant had offered to pay something towards the plaintiff's expenses.

"Mr. Serjeant PARRY submitted that there was no proof that the defendant had warranted the pills to cure the plaintiff, or that they were fit to be used by the plaintiff, nor that the defendant promised to give plaintiff skilful advice.

"The learned JUDGE thought the case must go to the jury.

"Mr. Serjeant PARRY then addressed the jury for the defendant. It was a very important matter to both parties, but particularly to the defendant, whose duty it was to take care that medicines were properly prepared, but not to give advice. The plaintiff had suffered through his own obstinacy in doctoring himself, and taking the advice of the beadle and his servant-maid, instead of going to his medical man. The defendant had sold the plaintiff what he asked for. Gout was not a complaint that obtained much sympathy, because it was supposed to arise from many hours of social enjoyment; but persons if they had the gout did not run after the parish beadle for advice, and then rush into the first chemist's shop and ask for the beadle's remedy. It would, however, be shown that the pills were perfectly innocent; they were prepared under the direction of Mr. Smith, the landlord of the Star and Garter tavern, in the King's-road, Chelsea, who had received great benefit from taking them. The plaintiff had asked for Smith's gout pills, but he had not told the assistant that he had been suffering from chronic gout. Smith's pills were given to him, and he took them home, and, concealing them from his medical attendant, he had taken advantage of the advice of the beadle, contrary to the express wish of his wife. The assistant would deny that he had told the plaintiff he might take three pills a day. Mr. Smith would be produced, for he was still alive, and believed it was through taking those pills that he was now alive. People who suffered from attacks of gout would run to any one for advice, and would take anything, and often double the quantity of anything, that was recommended to them. Mr. Smith had given the prescription, which was this—Calomel, 1 grain; colchicum, 1 grain; jalap, 1 grain; ipecacuanha, 1 grain. Lewty, the assistant of the defendant, had made up the pills, and each pill contained this quantity. Many medical men would state that it was a gout pill.

"Mr. Lewty said he had made up the pills. The plaintiff asked him for Smith's gout pills, and he gave him a box, and wrote upon the box, 'Two pills to be taken in a day.' He might have told him if two did not answer he might take three in a day. A person called some days afterwards, and the defendant said the pills only contained one grain of calomel in each pill. Never heard any one complain of the pills.

"Robert Alsopp, the predecessor of the defendant, stated that Smith had brought the prescription to his shop, and he sold them. There was a good demand for them. Never had any complaint about them.

"Mr. Smith became acquainted with the pills in 1850. He had the gout, and took the pills, and now suffered but little from that complaint. Recommended them to hundreds, but not to be taken as the plaintiff had taken them.

"Mr. Redwood, Professor of Chemistry, had analysed the pills, and found each pill weighed four grains, one of which was calomel.

"Dr. Andrew Barclay.—One of these pills taken twice a day was not injurious. They might be taken for the gout in suitable cases. Of course, he should inquire into the state and habits of a patient before prescribing for him. Mercury had a different effect upon different constitutions. Five grains taken within twenty hours would not be advisable. Calomel ought not to be taken without the person being watched by a medical man. One grain in some cases would produce salivation.

"Dr. W. Pettigrew.—This was a gout medicine, if taken carefully.

"Mr. W. Rendle, surgeon of thirty years' practice.—This was a good usual medicine, but persons should consult a medical man. The other ingredients would diminish the action of the mercury.

"The jury returned a verdict for the defendant."

The effect of counter practice upon the medical profession is simply to lower its members in the public estimation, and seriously to diminish the aggregate of their earnings—a well-deserved retribution, we think, for the original error of interfering at all with the supply and sale of drugs. But the result has been brought about by a very simple and easy process of ratiocination on the part of persons too ignorant or too idle to understand differences based upon education or degrees. Undeniable doctors sell physic—druggists sell physic: therefore druggists and doctors are all much the same thing. Indeed, the same course of reasoning has sometimes been carried a step farther. Druggists sell more physic, and keep more bottles, than their medical rivals; from whence it may be inferred that they know more—first, about the physic itself; secondly, about its uses. By the standard of value thus arrived at, not only physic vendors in the profession, but even physicians and pure surgeons are occasionally measured. We heard lately of a curious instance in which a patient consulted two physicians on the same morning, and then took their prescriptions to his druggist, and asked for advice as to which of the two should be prepared. The druggist suggested a compromise, and undertook to "put up" something which should combine the virtues of the two: thus constituting himself, in his self-sufficient ignorance, a referee between two gentlemen of education and ability. And not only, by means of counter practice, do the public suffer improper treatment, but the profession suffers absolutely, as well as relatively, in its reputation. The natural result of a trade competition with druggists has been to equalize the two callings by a very decided degradation of the superior one; and to credit medicine with much of the impudence, ignorance, and venality displayed from time to time by its irregular and ostensible followers.

The supply of drugs by medical practitioners has usually been defended on the ground that, in country districts especially, they could not otherwise be procured by the sick at all, and their dispensation and sale have been sources of considerable pecuniary profit. We regard these views as being wholly devoid of foundation.

It is true enough, probably, that the country druggist of a small town or large village, surrounded by surgeons who sell physic to their customers, will keep but a small stock of drugs and efficient preparations, and those often of inferior quality. The demand upon him for such things is small in absolute amount, and confined within very narrow limits in point of variety; and his customers are not possessed of sufficient knowledge to estimate the quality of his wares. If, however, his medical neighbours were to tell him that they intended to cease from dispensing, it would not only be to his interest and profit to keep all the preparations that they wished to use, but to keep them in such a state of purity and excellence as should ensure the fulfilment of the intentions of the prescribers. This is so obvious, we think, as hardly to admit of any question; and the only difficulty that could arise is from the possible insufficiency of the number of druggists now in business. We are disposed to believe, however, either that the present number would be sufficient, or that towns might often be weeded with advantage for the supply of adjacent country districts.

The question of profit to the profession is a more difficult one, and may be viewed in at least two aspects—first, as to the fact; and secondly, as to the amount of influence which it ought to exercise. We think the profit very questionable; and, moreover, that, if it can be shown to accrue, it may still be very dearly purchased.

In the first place, it is manifest that the pecuniary profit obtained by the sale of medicines must always represent a portion of what would be legitimately charged for advice. Under the system of practice which prevailed almost universally a few years ago, visits and home consultations were not charged at all, except to very wealthy patients, or in the case of visits to patients living beyond about a mile from the surgeon's house. The "doctor's bill" in all other cases was a mere enumeration of medicines supplied, and charged for at prices absurdly beyond their commercial value, in order indirectly to remunerate the vendor for his time and skill. Now that the custom of charging for visits has become almost universal, there is not only an enormous diminution in the quantity of medicine sent to the sick, but a reduction in the value set upon it, so that the general practitioner can scarcely charge more for a bottle of medicine than a druggist would do; scarcely more, that is to say, than its fair price as an article of trade. We have taken the pains to ascertain the value of the dispensing element in a practice conducted on this principle in the suburbs of London, and we found it to amount to about one-tenth of the total charges. For the sake of obtaining this, it is certainly not worth while to have the trouble of physic-making, the expense of materials and of assistance in dispensing, and the absolute loss entailed by bad debts. In places where visits would be charged at less than metropolitan rates, the price of medicine would bear a larger proportion to the whole; and with the less wealthy population, the same gross amount would represent a larger number of accounts, greater expense in the dispensing element, and a larger amount of bad debts; to say nothing of the confusion and trouble occasioned to the practitioner by the blending of a foreign element with his proper duties. The responsibilities of practice are quite enough, without adding to them those of physic-making; and we appeal confidently to the experience of our readers, whether this last be not the chief source of the disagreeables and worries of a doctor's life. If he have no assistant, a sudden call to a midwifery case will leave half his patients without their draughts and pills; while if he have an assistant, the carelessness and even unavoidable errors of that individual form ceaseless occasions of complaint. Either the promised blister was not sent at all, or it was sent at ten o'clock at night, or it was sent without any dressing; or there were no pills, or no directions on the pill-box; or the mixture was lighter or darker, sweeter or more sour, to-day than yesterday. Such are a few of the cries continually uttered by the patients of the dispensing surgeon; and all these matters have to be explained or apologized for by the man whose position ought to lift him above explanations and apologies, and whose mind ought to be left at liberty to concentrate itself upon its proper cares. This consideration alone—namely, that the sale of articles of trade places the medical practitioner in an inferior position to his patients among the higher classes, and renders him liable to their displeasure and censure for the neglect or imperfect performance of his functions in this respect —is, to our minds, a sufficient ground for the utter and unqualified condemnation of a system that bears such fruits. That a gentleman of liberal education and scientific attainments should be exposed to the chance of "losing a patient," or of otherwise suffering in purse or character, because he accidentally omitted to mix up a promised black draught for the pet attendant of some Lady Jones, is a state of things that ceases to be melancholy only because it is so ludicrous.

As in the case of most social abuses, however, it is difficult to fix blame anywhere but on "the system"—a system of gradual growth, and which can only be gradually overthrown. The regulations of the Apothecaries' Company, it is only fair to say, were not calculated to supply practitioners for whom physic-making would be an unsuitable form of industry; and it is but the growth of knowledge, and the general progress of society, that has produced improvements to which those regulations were, for many years, the greatest possible impediments, but which have followed upon their practical desuetude. The recent regulations of the Medical Council may be expected, year by year, to elevate the educational and social status of the general practitioner, and to make him less and less adapted to enter into trade relations with his patients, and the general abandonment of dispensing by the profession, the distinct disavowal of physic-making as a branch of medical duty, would at once break through the association which has made druggists, by virtue of their physic-making, *quasi* medical practitioners in the estimation of the public. If doctors made pills no longer, the position that the man who did make them must be a doctor would become one that could scarcely be maintained.

The next essential to which we have referred, is the placing of an authorized prescription within the reach of the poor.

As far as the wealthier and middle classes of society are concerned, the price of the medicine really necessary for the treatment of disease bears a proportion to a proper scale of charges for attendance that is so small as hardly to deserve the least consideration. A practitioner who does not seek to remunerate himself by medicine may study economy in his prescriptions, and may so arrange the doses and quantities ordered as to give very little expense to the patient: precisely as these doses and quantities were formerly so arranged as to give as much expense as possible. And it is obvious that a man who has no dispensing establishment may afford to deduct the cost of such an establishment from the gross amount of his charges; so that no patient need be called upon to pay more, to the doctor and druggist together, than he would do to the dispensing doctor alone.

But the provision of proper medical attendance upon the poor is a most perplexing and difficult matter to effect. When the late Mr. H. L. Smith, of Southam, brought his plan for provident dispensaries under the notice of the profession in London, it was objected that this difficulty is not ours to solve. "For the poor," remarked one speaker at a medical meeting, "proper attendance in sickness is as much a necessary as daily bread; and it is one which the State should provide, if the sick cannot. It is no more our duty to provide it, or to devise means by which it can be provided, than it is the duty of the bakers to supply the destitute with bread. Each baker pays his proportion to the poors' rate; and, if his bread be wanted for the poor, he receives its full value in exchange. So it should be with our skill." The speaker was much applauded, and his illustration was accepted as a pertinent and happy one. His hearers had sold physic until they had lost sight of the essential distinction between a medical man and a baker.

For we hold that the physician and the priest, members alike of the great body whose office it is to combat and mitigate, as much as may be possible, the evils, physical, moral, and spiritual, wrought by the dominion of the world, the flesh, and the devil, among mankind, should be the last to regard, in any merely sordid light, the sacred opportunities for good afforded by their respective callings. Truly, the labourer is worthy of his hire; but we have yet to see the instance in which conscientious labour failed to receive its reward.

Between the poor for whom provision is made during sickness by the State, and the classes able to pay for medical attendance, there is a wide margin of persons much exposed to illness or casualty, and only able to meet them by some kind of association during health. The associations for this purpose are far too much limited to the males, whose wives and families are perhaps the most frequent victims of "counter practice;" and it would be highly desirable, if possible, not only to break the seeming link between the druggist and the medical practitioner, but further, by the judicious formation and management of such associations, to do away with the temptation held out by the apparent cheapness of the druggist's shop. In a recent number (May 31, 1862) of the *British Medical Journal*, there is an admirable letter from the pen of Dr. Ogle, of Derby, on this very subject; although he takes "Hospital Abuses" as his text. Still he refers to "counter practice" in recommending that respectable druggists should be employed to dispense for the provident associations that he advocates; and we are disposed to think that the so-called "Hospital Abuses" are due to causes less upon the surface than those to which Dr. Ogle would ascribe them; and that they can be removed only by a far more thorough training for the duties of the profession than most of the licensing bodies at the present day either enforce or aspire to. Dr. Ogle suggests that the contributions of patients to the provident association should in every case be determined between the head of the family and the surgeon whom he may select: an arrangement that would admit many persons who are now excluded by an uniform low rate of charge, which is thought too little to be received from them. Dr. Ogle suggests various other regulations by which all, or nearly all, the objections to existing institutions of the kind might be removed, and by which a door would be opened for the exercise of a discriminating and wise charity towards the poor: that charity which it is at once the duty and the privilege of the profession to exhibit, and in the exhibition of which individuals are so sorely hindered, and their actions are so liable to misconstruction. There are few positions more difficult than that of a medical man who is called upon for a long attendance upon a patient to whom illness brings comparative poverty. The consequent debt is a pure misfortune to the invalid, who is unable even to exercise control over the amount to which it may rise, and who is therefore entitled to much consideration in his efforts to discharge it. On the other hand, the doctor owes it to his professional brethren to claim a fair remuneration for his services, and to take care that this claim is properly satisfied. The ordinary passive acquiescence of medical men in a large per centage of bad debts, exercises a decidedly demoralizing influence upon their patients, who too often postpone the payment of the doctor to every personal indulgence they may desire. From such evils as these, the associations ably advocated by Dr. Ogle would, we think, afford complete relief, the elasticity of the rule about payment enabling them to include families of widely different pecuniary circumstances. And moreover, their general prevalence would do much to promote the contract system of medical attendance among higher classes than any to which associations can appeal. Persons who would not make themselves "members" of anything, might yet pay a definite annual retaining fee to their medical attendant, of an amount to be agreed upon between them, so as to relieve him from all necessity for book-keeping, and to eliminate, as much as possible, the pecuniary element from professional intercourse. We can speak from personal experience of the advantages of this method for all concerned; and among them not the least is the establishment of mutual relations which bring the patient to the doctor at any time, without the fear of an indefinite bill before his eyes, and without any preliminary consultations across the counter of the druggist.

And finally, as the part which every individual can take in a needed reformation is to reform himself, so it is in the power of every medical practitioner to contribute to the abolition of "counter practice." Where the abandonment of dispensing is too decided a step to take, or where local difficulties may interfere with an adequate supply of drugs from other sources, something may still be done by the persistent endeavour to elevate the medical character, and to show its distinction from that of the trader. If barristers had been accustomed to seek payment for their opinions by a charge of guineas for the paper on which they were written, and if the number of guineas had been made to depend only on the size or number of the sheets, we have no doubt that the editor of the *Chemist and Druggist* would have applied, not to Mr. Chitty, but to some "counter practising" law stationer, for the document quoted at the commencement of this article. But the members of the Bar have kept themselves apart, socially and intellectually, from the tradesmen who supply the materials with which they work; and it is time that medical practitioners should do the same. The old distinction of classes within the profession is fast passing away; and it must be removed by the elevation of the general practitioner, not by the degradation of the physician.

